# Antral follicle count (AFC) and serum anti-Müllerian hormone (AMH) are the predictors of natural fecundability have similar trends irrespective of fertility status and menstrual characteristics among fertile and infertile women below the age of 40 years

**DOI:** 10.1186/s12958-019-0464-0

**Published:** 2019-02-11

**Authors:** Haroon Latif Khan, Shahzad Bhatti, Samina Suhail, Rohina Gul, Aisha Awais, Humaira Hamayun, Farah Enver, Sana Abbas, Zahira Hassan, Rameen Nisar, Saba Sardar, Warda Asif

**Affiliations:** 1 Lahore Institute of Fertility and Endocrinology, Hameed Latif Hospital, 14 New Abu Bakar Block Garden, 54800 Lahore, Pakistan; 2 Department of Human Genetics and Molecular biology, University of Health Sciences, Lahore, Pakistan; 3 Department of Medical Education, Rashid Latif Medical College, Lahore, Pakistan; 4 Department of Cellular Pathology, Royal Free Hospital, London, NW3 2QG UK; 5 Department of Biochemistry, Kinnard college for women, Lahore, Pakistan

**Keywords:** Antral follicle count, Anti-Müllerian hormone, Infertility, Ovarian reserve, ART

## Abstract

**Background:**

Despite being born with a significant number of primordial cells which representing the ancestor cells of the germ-line, women experience a depletion of ovarian reserve and sub-fertility mid-way into their healthy lives. The poor ovarian response is a substantial limiting factor amplified with higher maternal age and associated with a considerably lower likelihood of pregnancy.

**Methods:**

A present analytical prospective cross-sectional study was conducted to explore whether infertile women below the age of 40 years have low ovarian reserve than fertile women of same age, assessed by Antral follicle count (AFC) and anti-Müllerian hormone (AMH), at tertiary care infertility center: Lahore Institute of Fertility and Endocrinology, Hameed Latif Hospital. The study population including 423 infertile and 388 fertile female patients from June 2013 to November 2016. Patients and controls were aged between 25 and 39 years. Serum levels of FSH, LH, AMH were assessed, and AFC was measured by transvaginal sonography on cycle days 2 or 3.

**Results:**

A total of 35.6% of infertile women stated a menstrual cycle length shorter than 21 days, while 21% had a regular cycle length between 24 and 38 days, and 43.2%, longer than 38 days. Overall, the two cohorts did not significantly differ on cycle length. The age-specific reduction of the ovarian reserve was similar in both cohorts; serum AMH concentration decreased by 6% (95% Cl: 5–8%) and AFC decline by 4.5% (95% Cl: 5–7%) per year with increased age. Aged patients (36–39 years) had a 5.3% (95% Cl, 1.5; 7.2) higher risk ratio of having an AMH level < 0.7 ng/ml than women of younger age groups (Kruskal-Wallis test, *p* < 0.01).

**Conclusion:**

This study indicates that the possible common observation of low respondent in ART might not be a result of over-representation of patients with an early age-specific decline in the ovarian reserve, but rather primarily as a consequence of age-specific depletion in the stock of developing follicles at the time of recruitment and selection.

## Background

Over the past few decades, infertility has become increasingly problematic affecting 20–80 million people across the world. In well-developed countries, the proportion of couples seeking medical advice for infertility ranged from 3.5–16.7%, while in developing countries this range presented as 6.9–9.3% [1, 2].

Although several prospective population-based studies have investigated possible links between female age and infertility, however, the overall management of poor responders remains controversial during controlled ovarian stimulation [3]. The poor ovarian response is a substantial limiting factor amplified with higher maternal age and associated with a considerably lower likelihood of pregnancy. Moreover, advanced maternal age increased the risk of chromosomal abnormalities along with adverse maternal-perinatal outcomes such as fetal loss through miscarriages, obstetrical complications, severe maternal morbidities and coping with difficult management of pregnancy [4]. Iatrogenic procedures such as surgical removal of endometriomas and cysts are also responsible for the diminished ovarian reserve (DOR). Exclusively, genome-wide association studies have identified several linked loci of small genetic variations which determines the fetal antral follicle development and the progressive decline of the residual follicle pool over the course of the human reproductive span [5].

Endocrine variations among infertile patients primarily appear to be connected with the decline in feedback from extra- and intra-ovarian factors at the hypothalamic-pituitary-ovarian axis [6]. A gradual decline in the antral follicle pool in those with advanced maternal age firstly results in progressively elevated serum FSH concentration, followed by subsequent successive stages of irregular menstrual periods [7]. Furthermore, gradually decline in serum concentration of Anti-Müllerian hormone (AMH) is best characterized as a continuous reduction in the size of the antral follicle pool. The ovarian reserve can be evaluated by ovarian reserve tests such as detecting subsequent serum levels of AMH repeated measurements that describe changes in ovarian reserve over time and also through counting the resting basal antral follicles (AFC) which are assessed by transvaginal sonography. Both markers have appeared to be accurate in predicting feedback to control ovarian stimulation in the in-vitro fertilization setting [8, 9].

The onset of cycle irregularity, lack of or imbalance in certain hormones, poor ovarian reserve, and female reproductive age are the crucial factors which have a direct effect on the fertility of women [10]. Poor ovarian reserve is a complex clinical phenomenon often observed in women mainly in their mid to late thirties. Fifty-one is the average age to reach menopause with ages spanning from 46 to 62 years suggesting that the expected age-specific decline in the fertility fluctuates substantially between females of different ethnic origins [11].

Previous studies have revealed that women with low age-specific AMH might have had reduced early age-specific fertility that leads to both quantitative and qualitative deterioration in the oocyte complement resulting in a paradigm shift towards premature menopause [12].

Most of the early published reports reflect that infertile patients have a higher prevalence of early age-related decline of the follicles and they are the poor respondent of stimulation in ART. If this statement is considered correct, we would expect AMH and AFC lower in infertile women than fertile women of similar age groups. By considering these previous findings present study was conducted to explore to what extent impaired ovarian reserve contributes to infertility among infertile women below the age of 40 years.

## Methods

### Subjects

This study was designed as an analytical prospective cross-sectional cohort study. The study population included 423 infertile female patients who were referred between June 2013 to November 2016 for the assessment of ovarian and endocrine parameters at Lahore Institute of Fertility and Endocrinology, Hameed Latif Hospital. A total of 388 volunteer women were recruited in the control group. They have a regular menstrual cycle (length 24–38 days with five days difference between two consecutive cycles), no history of pelvic surgery and presence of both ovaries, have a normal ovarian reserve (did not meet the Bologna Criteria of low ovarian reserves). All control group participants are attending the Obstetrics and Gynaecology clinic for the following reasons: I) seeking for treatment of male partner due to erectile dysfunction II) counseling for preconception III) Contraceptive counseling and compliance III) request of tubal sterilization. Both patients and control group were aged between 25 and 39 years. The study was approved by the Institutional Ethical Committee (IEC) by Helsinki Declarations. The completed and signed consent form was obtained from all participants before research and publishing of the results of this study. Patients and control group were interviewed using a standardized registration form which included data entry for socio-economic status, age, BMI, genetic anomalies, demographic parameters, patient sexual history, and previous medical records. All participants were assured of high confidentiality. The patients and control were examined on cycle days 2 to 4.

### Inclusion criteria

Infertile women who were eligible for the study had the following criteria: I) no history of gynecological and abdominal surgery, II) having the normal sonographic texture of ovaries, and III) with no signs of hyper-androgenemia. Patients were stratified further into three different age categories 25–28, 29–35 and 36–39 respectively.

### Exclusion criteria

**Fig. 1:**
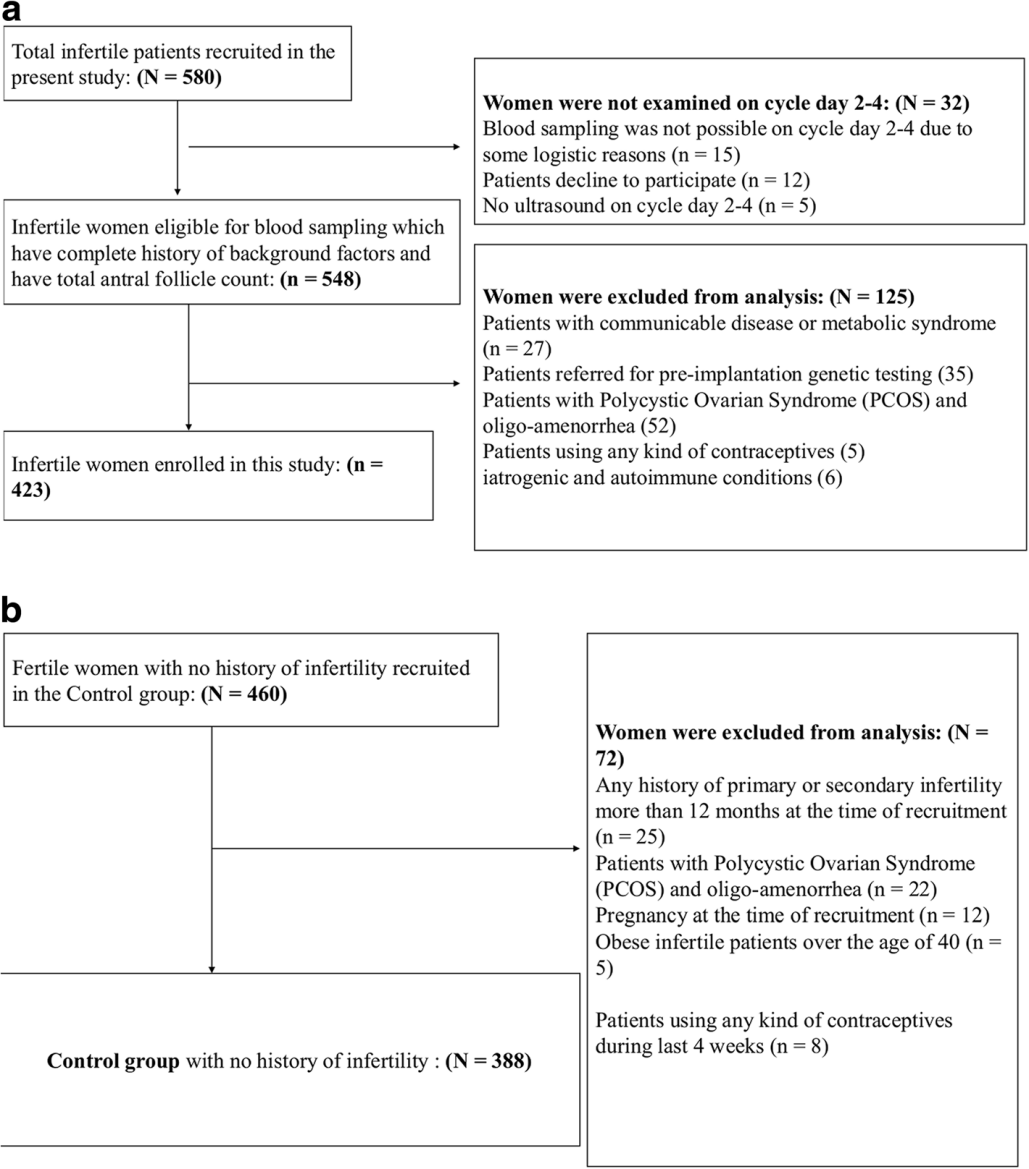
Study Flow chart of participants in the two cohorts. **a** Infertile patients recruited in the prospective cross-sectional study. **b** Control group with no history of infertility

The following patient cohorts were excluded from the study: I) those having any communicable disease or metabolic syndrome, II) patients referred for pre-implantation genetic testing, III) patients with Polycystic Ovarian Syndrome (PCOS) and oligo-amenorrhea, IV) patients using any contraceptives, V) those having iatrogenic and autoimmune conditions VI) Obese infertile patients over the age of 40. Figure 1 provides the flow chart of the study population.

### Pelvic ultrasonography

A transvaginal ultrasound was performed on day 2 or 3 of their cycle. The ovarian volume was evaluated as previously defined by Rosendahl et al. (2010). Diagnosis of ovarian cysts, fibromas, and endometriomas was made accordingly. Antral follicles were counted and grouped according to their sizes 2 – 5 mm (small), 5 – 8 mm (intermediate) and 8 – 10 mm (large).

## Evaluation of endocrine dimensions

Blood samples were collected between 8 to 10 am from a cubital vein on cycle day 2–4 before starting the controlled ovarian stimulation protocols. Serum was separated and kept refrigerated at − 20 °C until the routine hormonal analysis was performed, that included FSH, AMH, and LH through electrochemiluminescence immunoassay according to the manufacturer’s instructions (Elecsys® Roche Diagnostics, Indianapolis, USA). The analytical sensitivity, intra and inter-assay coefficient of variation for FSH was < 0.2 IU/I, 2.3 and 4.2% (95% Cl) and < 0.02 IU/ml 1.3 and 2.5% (95% Cl) for LH respectively. The level of AMH was in the range of 0.01 to 23 ng/ml with an internal coefficient of variation 1.8% for repeatability and 4.4% for intermediate precision by Gen II ELISA (Calibrator and Controls, RUO USA).

## Statistical analysis

Patients were categorized into three different age groups 25–28, 29–35, 36–39 and baseline characteristics were calculated as means ± SD, number percentage [n%] and median with 95% population limits as applicable. The difference between patient and control groups as well as age-specific subgroups was determined through one-way ANOVA for normally distributed continuous variable data. However, a Kruskal-Wallis / two-tailed test was performed to find the outcome differences in the distribution of values across the three age groups. χ^2^-test was used for the analysis of categorical variables. The mean difference between the different follicles size was calculated by permutation tests, whereas the mean confidence interval and *p*-values were calculated by bootstrapping (R version 3.4.4).

Furthermore, the natural logarithmic transformation for serum concentrations of AMH and AFC was employed for further calculations. Spearman’s rank correlation coefficient was used to explore the relationships among levels of AMH and AFC. Scatter plots were drawn to find the age-specific changes based on ovarian reserve parameters in the patient versus the control group. A linear regression analysis was performed to highlight whether the age-specific decline accelerated with the progression of age between the patient and control groups after adjusting age as a continuous variable. The comparison between the prevalence of low ovarian reserves among two cohorts was tested through logistic regression analysis. For lower ovarian reserves the optimal lower cut-off level of AMH was set at 0.72 ng/ml [13], and upper cut off level was 8 ng/ml (95% Cl) along with AFC < 8, as proposed by Ferraretti and Glanaroli (2011). Statistical analysis including descriptive analysis was performed using statistical package SPSS (version 25; SPSS Inch., Chicago, IL, USA) and XLSTAT 2017 software. A *p*-value less than 0.05 was considered as statistically significant.

## Results

### Demographic and socioeconomic characteristics

The distribution of demographic characteristics of patient and control groups were given in Table 1. The mean age was not significantly different between infertile patients and the control group (29.9 ± 4.10 versus 30.41 ± 3.9; *p* = 0.6). Although the two cohorts shared similar characteristics regarding BMI and occupation, a greater number of infertile patients had a history of anemia compared with the controls (54.1% versus 41.7%: *p* < 0.001). In comparison with control group, fewer infertile women reported having a moderate (30.2% versus 36.9%: *p* < 0.001) to intense (16.7 versus 39.1%; p < 0.001) physical activity. For the duration of physical activity, there was 22.7% decreased the risk of subfertility in those who had a moderate mode of exercise (2–6 h/week) compared with the shortest duration of fewer than 2 h/week. The risk of infertility was not associated with caffeine consumption in moderate consumers with a hazard ratio 1.09 (95% Cl: 0.95–1.08) and intense consumers with a hazard ratio 1.05 (95% Cl: 0.98–1.09) than the controls 1.02 (95% Cl: 0.89–1.03).

**Table 1:** Demographic and clinical parameters of the study population comprising 423 infertile and 388 fertile women

Parameters	Infertile Patient Age groups (years)					Control Age groups (years)					Infertile vs. controls *p*- value
	25–28	29–35	36–39	*p*- value	Total	25–28	29–35	36–39	*p*- value	Total	
Number of women [n (%)]	90 (21.2)	210 (49.6)	123 (29.07)	–	423 (100)	101 (26.01)	165 (42.5)	122 (31.4)	–	388 (100)	–
Age (years) [mean ± SD]	26.8 ± 1.1	32.6 ± 1.7	37.9 ± 1.4	_	29.9 ± 4.10	26.94 ± 1.8	33.13 ± 1.9	38.15 ± 1.7		30.41 ± 3.9	0.6^a^
BMI [median (95% population limit)]	26.4 (17.4;26.9)	27 (19.1;27.8)	27.6 (20.1;36.9)	0.08^b^	25.3 (18.1;29.9)	26.3 (19.1;29.5)	26.9 (18.9;31.1)	28.1 (18.4;31.9)	0.03^b^	24.9 (18.2;30.1)	0.8^b^
Previously conceived [*n* (%)]	32 (35.5)	98 (46.6)	68 (55.2)	0.71^b^	198 (46.8)	84 (83.1)	112 (67.8)	98 (80.3)	0.001	294 (75.7)	< 0.001
Any history of Anemia [*n* (%)]											
Present	69 (76.6)	112 (53.3)	48 (39)	0.002^c^	229 (54.1)	29 (28.7)	55 (33.3)	78 (63.9)	< 0.001^c^	162 (41.7)	< 0.001^c^
Absent	21 (23.3)	98 (46.6)	75 (609)		194 (45.8)	72 (71.2)	110 (66.6)	44 (36.06)		226 (58.2)	
Physical Activity [*n* (%)]											
Low (< 2 h/week)	43 (47.7)	112 (53.3)	69 (56)	< 0.001^c^	224 (52.9)	23 (22.7)	36 (21.8)	23 (18.8)	< 0.001^c^	82 (21.1)	< 0.001^c^
Moderate (2–6 h/week)	25 (27.7)	78 (37.1)	25 (20.3)		128 (30.2)	33 (32.6)	78 (47.2)	43 (35.2)		154 (36.9)	
Intense (> 6 h/week)	22 (24.4)	20 (9.5)	29 (23.5)		71 (16.7)	45 (44.5)	51 (30.9)	56 (45.9)		152 (39.1)	
Caffeine consumption (mg/day) [*n* (%)]											
Non- consumers	40 (44.4)	98 (46.6)	66 (53.6)	< 0.001^c^	204 (48.2)	61 (60.3)	75 (45.4)	48 (39.3)	< 0.002^c^	184 (47.4)	0.3^c^
Moderate consumers (100–300)	30 (33.3)	76 (36.1)	41 (33.3)		147 (34.7)	21 (20.7)	51 (30.9)	31 (25.4)		103 (26.5)	
Intense consumers (> 300)	20 (22.2)	36 (17.1)	16 (13)		72 (17)	19 (18.8)	39 (23.6)	43 (35.2)		101 (26)	
Occupation [*n* (%)]											
House wife	15 (16.6)	85 (40.4)	39 (31.7)	0.5^c^	139 (32.8)	43 (42.5)	76 (46)	47 (38.5)	0.6^c^	166 (42.7)	0.5^c^
Business women	25 (27.7)	45 (21.4)	13 (10.5)		83 (19.6)	5 (4.9)	12 (7.2)	9 (7.3)		26 (6.7)	
Govt. employees	15 (16.6)	10 (4.7)	8 (6.5)		33 (7.8)	3 (2.9)	9 (5.4)	6 (4.9)		18 (4.6)	
Others	35 (38.8)	70 (33.3)	63 (51.2)		168 (39.7)	50 (49.5)	68 (41.2)	58 (47.5)		176 (45.3)	
Monthly Income (Rupees) [*n* (%)]											
Low income 30,000-50,000	5 (5.5)	10 (4.7)	16 (13)	< 0.001^c^	31 (7.3)	28 (27.7)	35 (21.1)	31 (25.4)	< 0.001^c^	94 (24.2)	0.1^c^
Moderate income 50,000- 100,000	25 (27.7)	38 (18)	21 (17)		84 (19.8)	39 (38.6)	59 (35.7)	45 (36.8)		143 (36.8)	
High income > 100,000	60 (66.6)	162 (77.1)	86 (69.9)		308 (72.8)	34 (33.6)	71 (43)	46 (37.7)		151 (38.9)	

Data are presented as number [%age], mean ± SD and median [95% population limits]. For both patients and controls, data are classified into age-specific sub-groups, and a statistical difference was tested across different age groups. Data for the total infertile and controls are also subjected to statistical differences, and *p* > 0.05 considered statistically significant

^a^= One-way ANOVA; ^b^ = Kruskal-Wallis test; ^c^ = χ^2^-test

### Reproductive and menstrual characteristics

Typically, in females, the first menstrual cycle occurs around 2 to 2.5 years after the appearance of breast buds. In the present study, the median age at menarche was 13.5 years in infertile patients (95% Cl: 11–16.1 years). For the whole cohort, the mean baseline age at menarche was 12.8 ± 1.5 years in the infertile group and 12.6 ± 1.26 years in the control group respectively. Overall, 87% of infertile women experienced menarche below the age of 14, while the remaining 13% reported an age greater than 15 years. A similar relationship was observed in 94.5% of control subjects where the age at menarche was under 14, and 5.5% observed at the age of 15 years. There were no statistically significant differences in the onset of early (≤ 11 years) and late age (≥ 15) menarche between infertile patients and fertile controls (95% Cl: 11–16.1 years; *p* = 0.09). However, the frequency of early menarche was higher in infertile women (19.5%) aged 36–39 years compared to fertile controls (11.4%) and other age-specific subgroups of patients (Table 2). A total of 35.6% of infertile women stated a menstrual cycle length shorter than 21 days, while 21% had a regular cycle length between 24 and 38 days, and 43.2% had cycle length longer than 38 days. Overall, the two cohorts significantly differ on cycle length (*p* < 0.01). Based on the average duration of menstrual flow in a cycle, 28.1% of infertile women had prolonged menstruation (> 8 days), and 56.2% reported shorter periods (< 4 days). The proportion of patients who had no bleeding in a cycle was higher over the age of 36 (30.8%; *n* = 123, *p* < 0.001) as compared to controls. Whereas 15.6% percent of women reporting menses last for 4–8 days.

**Table 2:** Menstrual characteristics in women under the age of 40 years

Parameters	Infertile Patient Age groups (years)					Control Age groups (years)					Infertile vs. controls *p*-value
	25–28	29–35	36–39	*p*- value	Total	25–28	29–35	36–39	*p*- value	Total	
Number of women [*n* (%)]	90 (21.2)	210 (49.6)	123 (29.07)	–	423 (100)	101 (26.01)	165 (42.5)	122 (31.4)	–	388 (100)	–
Age at menarche (years) *n* (%)]											
≤ 11	12 (3.3)	35 (16.6)	24 (19.5)	0.1^c^	71 (16.7)	8 (7.9)	11 (6.6)	14 (11.4)	0.2^c^	33 (8.5)	0.09^c^
12–13	49 (54.4)	108 (51.4)	59 (47.9)		216 (51)	79 (78.2)	98 (59.3)	23 (18.8)		200 (51.5)	
13–14	10 (11.1)	45 (25.7)	31 (14.7)		86 (20.3)	12 (11.8)	41 (24.8)	81 (66.3)		134 (34.5)	
≥ 15	19 (21.1)	22 (10.4)	9 (7.3)		50 (11.8)	2 (1.9)	15 (9)	4 (3.2)		21 (5.4)	
Menstrual Cycle length (days) [mean ± SD]	33.4 ± 3.1	29.9 ± 1.9	28.8 ± 1.8	0.4^a^	30.2 ± 2.0	28.9 ± 2.1	29.1 ± 2.3	30.2 ± 1.9	0.3^a^	29.9 ± 2.1	0.5^a^
Frequency of Menses (days) [n (%)]											
Frequent (< 21)	31 (34.4)	85 (40.4)	35 (28.4)	< 0.001^c^	151 (35.6)	1 (0.99)	0 (0.00)	1 (0.08)	< 0.001^c^	2 (0.51)	0.01^c^
Normal (24–38)	14 (15.5)	46 (21.9	29 (23.5)		89 (21)	100 (99.0)	164 (99.3)	121 (99.1)		385 (99.2)	
Infrequent (> 38)	45 (50)	79 (37.6)	59 (47.9)		183 (43.2)	0 (0)	1 (0.6)	0 (0)		1 (0.25)	
Cycle to Cycle variation over 12 months (days) [n (%)]											
Regular (variation ±2–20 days)	15 (16.6)	29 (13.8)	14 (11.3)	< 0.002^c^	58 (13.7)	101 (100)	165 (100)	121 (99.1)	< 0.001^c^	387 (99.8)	0.03^c^
Irregular (variations > 20 days)	75 (83.3)	190 (90.4)	109 (88.6)		374 (88.4)	0 (0)	0 (0)	1 (0)		1 (0.25)	
Duration of blood flow (days) [n (%)]in one episode											
Prolonged (> 8)	23 (25.5)	65 (30.9)	31 (25.2)	< 0.001^c^	119 (28.1)	19 (18.8)	35 (21.2)	15 (12.2)	< 0.001^c^	69 (17.7)	0.2^c^
Normal (4–8)	12 (3.3)	35 (16.6)	19 (15.4)		66 (15.6)	53 (79.2)	80 (48.4)	62 (50.8)		195 (50.2)	
Shortened (< 4)	55 (61.1)	110 (52.3)	73 (59.3)		238 (56.2)	29 (28.7)	50 (30.3)	45 (36.8)		123 (31.7)	
Monthly blood loss (ml) [*n* (%)]											
Light (< 5)	23 (25.5)	73 (34.7)	15 (12.1)	< 0.001^c^	111 (26.2)	18 (17.8)	22 (13.3)	60 (49.1)	< 0.001^c^	100 (25.7)	0.3^c^
Normal (5–80)	25 (27.7)	49 (23.3)	10 (8.1)		84 (19.8)	55 (54.4)	101 (61.2)	52 (42.6)		208 (53.6)	
Heavy (> 80)	20 (22.2)	63 (30)	36 (29.2)		119 (28.1)	25 (24.7)	40 (24.2)	10 (8.1)		137 (35.3)	
No bleeding	22 (24.4)	25 (11.9)	38 (30.8)		85 (20)	0 (0)	0 (0)	0 (0)		0 (0)	
Occurrence of Blood clots [*n* (%)]											
Yes	26 (28.8)	98 (46.6)	48 (39)	< 0.001^c^	172 (40.6)	59 (5.8)	129 (78.1)	66 (54)	< 0.001^c^	254 (65.4)	0.07^c^
No	64 (71.1)	112 (53.3)	75 (60.9)		251 (59.3)	31 (30.6)	36 (21.8)	56 (45.9)		123 (31.7)	
Former history of abortions [*n* (%)]											
0	81 (90)	191 (90.9)	118 (95.9)	< 0.001^c^	390 (92.1)	97 (96)	158 (95.7)	110 (90.1)	< 0.001^c^	365 (94)	0.2^c^
≥ 1	9 (10)	19 (9)	5 (4.06)		33 (7.8)	4 (3.9)	7 (4.2)	12 (9.8)		23 (5.9)	

Data are presented as number [%age], mean ± SD and median [95% population limits]. For both patients and controls, data are classified into age-specific sub-groups, and a statistical difference was tested across different age groups. Data for the total infertile and controls are also subjected to statistical differences, and p > 0.05 considered statistically significant

^a^= One-way ANOVA; ^c^ = χ^2^-test

In an age-specific subgroup analysis, restricted to infertile women aged 29–35 years, heavy bleeding (> 80 ml) was reported 30% while other two subgroups reported 22.2% (25–28 years) and 29.2% (36–39 years) respectively. Those patients who reported blood clots during menstruation tend to have prolonged menstruation (95% Cl; 9.6–15.4) than those who reported no blood clots.

### Evaluation of ovarian reserve based on ultrasound findings and hormonal parameters in age-specific subgroups of patients and controls

**Fig. 2:**
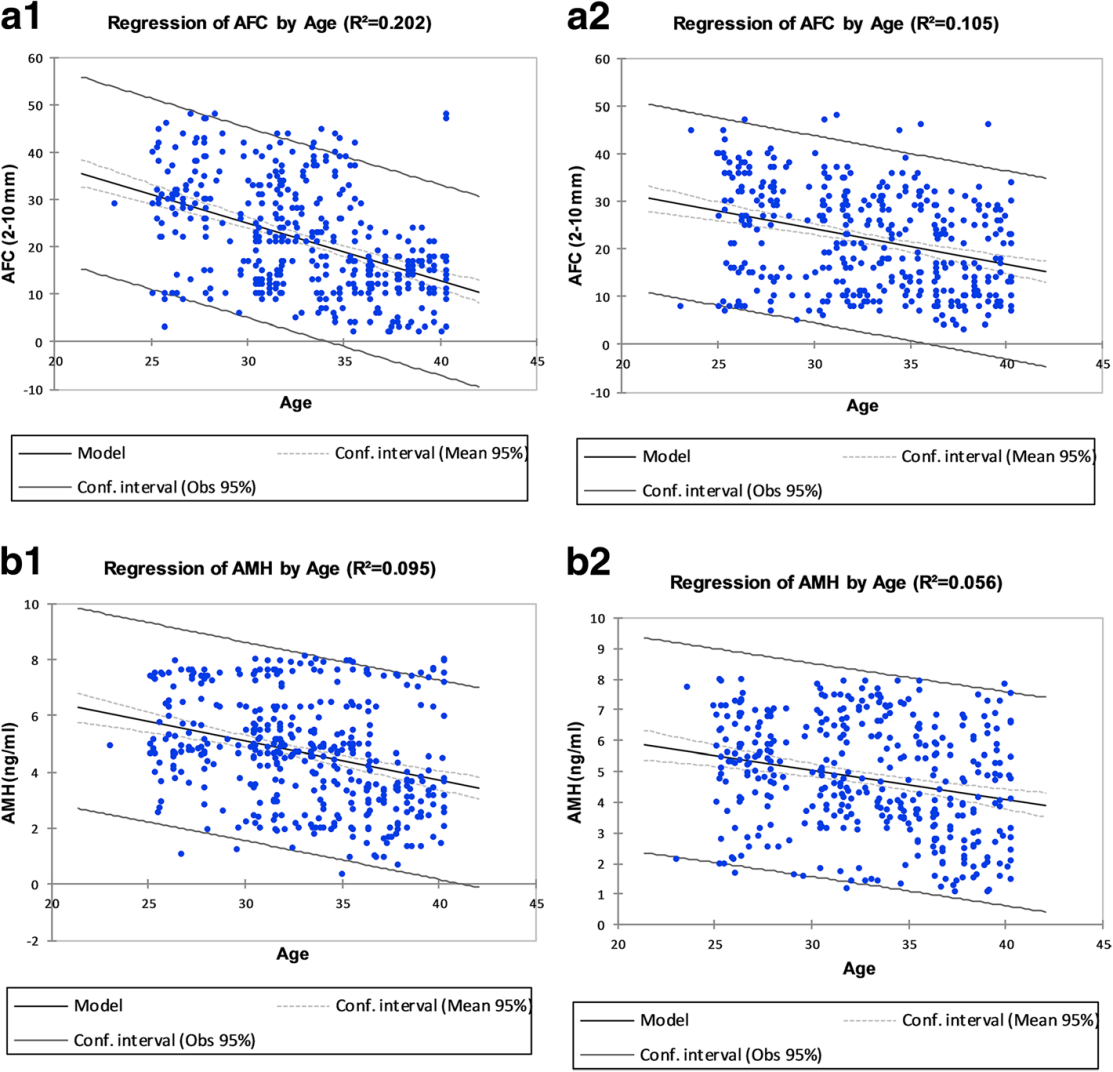
Association between ovarian reserve markers and age of the patients and control groups. **A1**) Total antral follicle count (2–10 mm) 1) in 423 Infertile patients. **A2**) 388 control patients of the same age displayed total antral follicle count below the age of 40. **B1**) Serum AMH concentration in 423 infertile patients below the age of 40. **B2**) 388 controls with no history of infertility showed serum AMH concentration under the age of 40. The dotted lines characterize 95% confidence limits

Infertile patients revealed significantly higher serum concentration of FSH (95% Cl; 7.6; 8.4, *p* < 0.01), LH (95% Cl: 6.1; 6.8, *p* = 0.03) and a raised LH/FSH ratio (95% Cl; 0.7; 1.6, *p* < 0.01) than controls as displayed in the Table 3. Moreover, after controlling suspected confounding factors such as age serum FSH concentration remain elevated among patient groups (7, 95%Cl: 2:11%). As shown in Fig. 2, the age-specific reduction of ovarian reserves was similar in both cohorts; serum AMH concentration decreased by 6% (95% Cl: 5–8%) and AFC decline by 4.5% (95% Cl: 5–7%) per year with an increased age.

**Table 3:** Hormonal and ultrasonographic characteristics of the study cohorts evaluated on cycle day 2 to 4

Parameters	Infertile Patient Age Group (years)					Control Age Group (years)					Infertile versus control *p*-value
	25–28	29–35	36–39	*p*-value	Total	25–28	29–35	36–39	*p*-value	Total	
Number of women [*n* (%)]	90 (21.3)	210 (49.6)	123 (29.07)		423 (100)	101 (26.0)	165 (42.5)	122 (31.4)		388 (100)	
Endocrine parameters											
AMH (ng/ml) [median (95% population limit)]	5.1 (3.2;9.9)	4.8 (4.5;8.0)	3.4 (2.5;7.1)	< 0.002^b^	4.8 (4.5;6.9)	5.4 (4.9;9.6)	5.0 (4.9;9.4)	3.6 (3.6;9.2)	< 0.001^b^	5.0 (3.2;6.8)	0.08^b^
AMH (ng/ml) categories [n (%)]											
< 0.7	3 (3.3)	28 (13.3)	25 (20.3)	0.002^c^	56 (13.2)	4 (3.96)	12 (7.3)	18 (14.7)	< 0.001^c^	34 (8.7)	0.3^c^
0.7–4.5	16 (17.7)	45 (21.4)	65 (52.8)		126 (29.7)	15 (14.9)	38 (23.0)	52 (42.6)		105 (27.0)	
4.5–7.9	45 (50)	107 (50.9)	18 (14.6)		170 (40.1)	68 (67.3)	85 (51.5)	47 (38.5)		200 (51.5)	
≥ 8.0	26 (28.9)	30 (14.3)	15 (12.2)		71 (16.7)	14 (13.9)	30 (18.1)	5 (4.0)		49 (12.6)	
FSH (IU/L) [median (95% population limit)]	6.9 (6.5;8.1)	7.1 (7.1;8.3)	7.2 (8.1;9.5)	0.3^b^	7.1 (7.6;8.4)	6.5 (5.5;6.8)	6.7 (5.9;7.2)	6.8 (5.8;7.0)	0.8^b^	6.6 (6.0;7.1)	0.01^b^
FSH (IU/L) categories [*n* (%)]											
< 4	17 (18.8)	22 (10.5)	3 (2.4)	0.4^c^	42 (9.92)	5 (4.9)	11 (6.6)	4 (3.3)	0.5^c^	20 (5.1)	0.07^c^
5–8	58 (64.4)	158 (75.2)	65 (52.8)		281 (66.4)	45 (44.5)	75 (45.5)	41 (36.0)		161 (41.5)	
9–14	10 (11.1)	9 (4.3)	35 (28.5)		54 (12.7)	35 (34.6)	45 (27.2)	69 (56.5)		149 (38.4)	
15–20	5 (5.5)	21 (10)	20 (16.3)		46 (10.8)	16 (15.8)	34 (26.6)	8 (6.5)		58 (14.9)	
LH (IU/L) [median (95% population limit)]	6.4 (6.0;7.1)	6.3 (5.8;6.7)	6.5 (6.1;7.0)	0.7^b^	6.3 (6.1;6.8)	5.9 (5.5;6.3)	5.5 (4.4;5.9)	5.4 (5.1;6.7)	0.07^b^	5.5 (5.1;7.1)	0.03^b^
LH/FSH-ratio (IU/L) [median (95% population limit)]	1 (0.8;1.8)	0.9 (0.6;1.7)	0.9 (0.8;1.5)	0.4^b^	0.9 (0.7;1.6)	0.8 (0.5;1.9)	0.8 (0.4;1.5)	0.8 (0.6;1.8)	0.7^b^	0.8 (0.5;1.7)	0.01^b^
Ultrasonography parameters											
Total number of AFC [median (95% population limit)]	30 (26.9;31.5)	22 (21.7;24.6)	14 (12;14.2)	< 0.0001^b^	21 (20.5;28.6)	29.0 (24.7;29.0)	23.0 (24.7;29.0)	14.0 (16.4;20.5)	< 0.001^b^		0.7^b^
AFC categories [n (%)]											
< 8	0 (0)	14 (6.7)	25 (20.3)	< 0.0001^c^	39 (9.2)	0 (0)	2 (1.2)	8 (6.5)	0.0001^c^	10 (2.5)	0.7^c^
9–20	15 (16.7)	71 (3.3)	88 (71.5)		174 (41.1)	28 (27.7)	66 (40)	70 (57.3)		164 (42.2)	
21–45	69 (76.7)	117 (55.7)	8 (6.5)		194 (45.8)	68 (67.3)	93 (56.3)	43 (35.2)		204 (52.5)	
> 46	6 (6.7)	8 (3.8)	2 (1.6)		16 (3.78)	5 (4.9)	4 (2.4)	1 (0.8)		10 (2.5)	
Ovarian volume (ml) [median (95% population limit)]	5.6 (3.2;11.6)	5.4 (2.5;10.6)	5.3 (2.3;11.9)	0.2^b^	5.5 (2.5;11.7)	5.1 (2.1; 8.97)	4.9 (2.7;9.1)	4.5 (2.3;9.9)	0.001^b^	4.9 (2.3;8.9)	0.008^b^

Data are presented as number [%age] and median [95% population limits]. For both patients and controls, data are classified into age-specific sub-groups, and a statistical difference was tested across different age groups. Data for the total infertile and controls are also subjected to statistical differences, and *p* > 0.05 considered statistically significant

^b^ = Kruskal-Wallis test; ^c^ = χ^2^-test

**Fig. 3:**
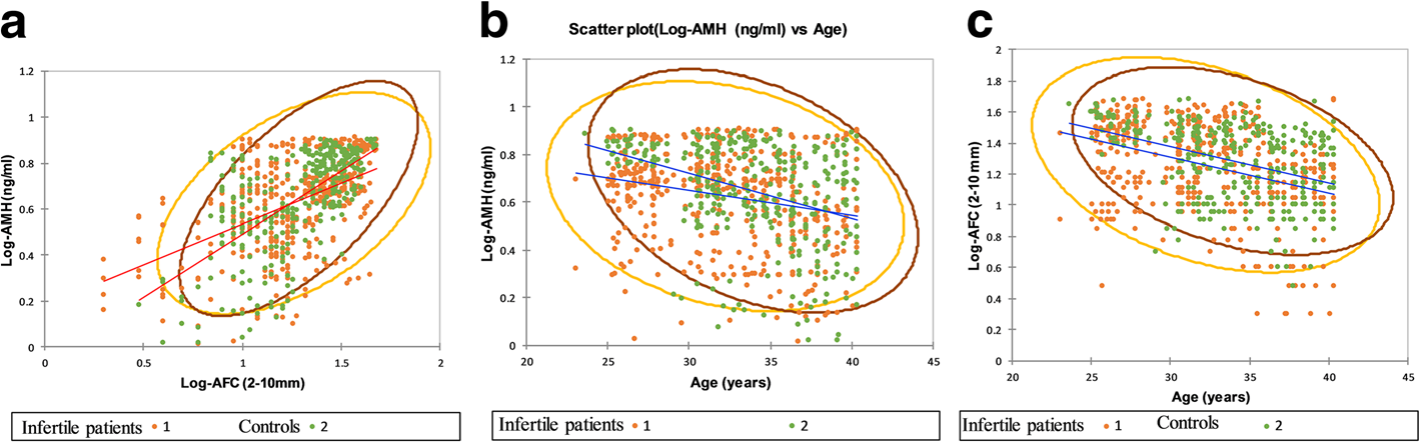
Scattered plots represent the linear association between ovarian reserve markers and age in infertile patients versus controls. **a**) Correlation between AMH and AFC in infertile patients (*n* = 423, r^2^ = 0.83, *P* < 0.001), controls (*n* = 388, r^2^ = 0.82, P < 0.001). **b** The age-specific decline in AMH in two cohorts. **c** The age-specific decline in AFC in two cohorts

Figure 3a depicted a linear correlation between serum AMH concentration and AFC (for the total samples: Spearman’s rank correlation coefficient = 0.91, *p* < 0.002). As demonstrated in Fig. 3b and c, after adjustment for age neither AMH (12, 95% Cl: − 2; 26) nor AFC (3, 95% Cl; − 8; 10%) were significantly different in both cohorts. The same scenario was observed in the two cohorts after adjustment for BMI, and caffeine consumption (AMH: 9, 95% Cl: − 8; 25 and AFC: 1, 95% Cl; − 7; 8%). However, in the infertile subjects, ratio of non-dominant small follicles was significantly lower (6%, 95 Cl: 2.1; 5.6%, *p* < 0.001) and percentage of large follicles was significantly higher than controls (4, 95% Cl: 2.3; 4.6%, p < 0.001) (Table 4).

**Table 4:** Mean proportion of antral follicles among different age-specific groups of infertile patients and controls

Age Groups	Infertile patient Age groups			Control Age groups			Mean difference (95% Cl)	Infertile versus control *p*-value
	25–28	29–35	36–39	25–28	29–35	36–39		
No of samples *n* (%)	90 (21.2)	210 (49.6)	123 (29.07)	101 (26.03)	165 (42.5)	122 (31.4)		
Total AFC [median (95% population limits)]	27.1 (23.9;30.1)	23.0 (22.3;25.2)	17.1 (15.3;19.9)	28.9 (24.8;31.2)	23.4 (19.7;25.6)	16.9 (14.9;19.8)	−0.29 (−2.01;1.33)	0.6
AFC (mm), mean proportion (95% population limits)								
AFC 2–5	0.71 (0.7;0.72)	0.68 (0.61;0.76)	0.61 (0.59;0.72)	0.8 (0.71;0.79)	0.76 (0.61;0.72)	0.68 (0.64;0.69)	−0.06 (− 0.09; −0.03)	< 0.001
AFC 5–8	0.41 (0.37;0.36)	0.42 (0.38;0.44)	0.44 (0.39;0.41)	0.31 (0.28;0.32)	0.3 (0.29;0.32)	0.3 (0.26;0.32)	0.03 (−0.005;0.054)	0.2
AFC 8–10	0.06 (0.03;0.08)	0.07 (0.05;0.08)	0.08 (0.07;0.98)	0.04 (0.03;0.04)	0.04 (0.03;0.05)	0.05 (0.05;0.07)	0.04 (0.03;0.05)	< 0.001

Data are presented as [median (95% population limits)] and number [%age]. Premutation tests were used to estimate the mean differences. P-values were calculated through bootstrapping and *p* > 0.05 considered statistically significant

### Prevalence of poor ovarian reserves

A deficit tendency of serum AMH (< 0.7 ng/ml) was observed in 13.2% of patients compared with 5.1% of controls (age-adjusted odds ratio model: 1.0, 95% Cl: 0.5; 2.01), that was eminently age-dependent. Aged patients (36–39 years) had a 5.3% (95% Cl: 1.5; 7.2) higher risk ratio of having an AMH level < 0.7 ng/ml than women of younger age groups (Kruskal-Wallis test, *p* < 0.01). We also observed that AMH levels < 4.5 ng/ml were less common in infertile women (15, 95% Cl: 0.5; 3.6) than controls (20.3, 95% Cl: 0.6; 3.7). Whereas the ratio of AFC < 8 was not significantly different between the two cohorts (*p* = 0.7) (Table 2). Regardless of the cut-off level employed, the domination of poor ovarian reserve was similar amongst the two cohorts after adjustment for BMI, education, caffeine consumption. Patients with intense physical activity and non-smokers tend to have a low prevalence of having an AMH < 0.7 ng/ml.

### Ovarian reserve markers and infertility investigation

The most common cause of infertility was the high contribution of male-related factors and unexplained infertility (Table 5). The prevalence of male infertility was similar in age-specific subgroups of below 35 years (35.5 and 35.7%) and above 36 years (39.02%). Interestingly, 30% of male infertility was linked with the higher serum concentration of AMH in patients aged 29–35 years (Cl: 6; 58%) and 38% was associated for patients aged 36–39 years (95% Cl: 10;92%). Whereas, AFC was associated with 19% (95% Cl: 5; 42%) for patients aged 29–35 years and 28% (95%Cl: 8; 62%) for patients aged 36–39 years respectively. However, patients aged 25–28 years had no difference in the serum concentration of AMH and AFC with or without male infertility (data was not shown). After the exclusion of male infertility factors the serum concentration of AMH (3, 95%Cl: − 9;19%) and AFC (− 8, 95% Cl: − 16;29%) remained similar between the two cohorts. As seen in Table 5, the distribution of unexplained infertility was higher (18.8%) in women aged 25–28 years than other age groups of patients (7.1 and 11.3%). Patients who reported with unexplained infertility had similar serum concentration of AMH (− 9, 95% Cl: − 19; 12%) and AFC after adjustment for age (− 6, 95% Cl: − 15;9%) compared with other age-specific subgroups analysis (Kruskal-Wallis test).

**Table 5:** Infertility Diagnosis and duration for an age-specific total cohort of infertile women

Parameters	Age groups			
	25–28	29–35	36–39	Total
No of samples [*n* (%)]	90 (21.2)	210 (49.6)	123 (29.07)	423 (100)
Infertility duration (months) [median (95% population limits)]	28 (15;42)	31 (10;60)	35 (15;61)	29 (11;58)
Primary infertility [*n* (%)]	78 (86.6)	85 (40.4)	57 (46.3)	220 (52.0)
Secondary infertility [*n* (%)]	12 (13.3)	125 (59.5)	166 (134.9)	303 (71.6)
History of fertility treatment [*n* (%)]	20 (22.2)	198 (94.3)	110 (89.4)	328 (77.5)
Female factors [*n* (%)]				
Anovulation	2 (2.2)	3 (1.4)	1 (0.8)	6 (1.4)
Endometriosis	3 (3.3)	5 (2.5)	4 (3.3)	12 (2.8)
Tubal Blockage	6 (6.6)	8 (3.8)	4 (3.2)	18 (4.2)
Multiple female factor	12 (13.3)	20 (9.5)	8 (6.5)	40 (9.5)
Sub-mucous myomas	8 (8.8)	28 (13.3)	7 (5.7)	43 (10.2)
Laparotomy	3 (3.3)	5 (2.4)	1 (0.8)	9 (2.1)
Male factor [*n* (%)]				
Male infertility	32 (35.5)	75 (35.7)	48 (39.02)	155 (36.6)
Both Male and Female factor	7 (7.7)	49 (23.3)	36 (29.2)	92 (21.7)
Unexplained infertility factors	17 (18.8)	15 (7.1)	14 (11.3)	46 (10.8)

Data are presented as number [%age] and median [95% population limits]

## Discussion

Tracking the early decline ovarian reserve and loss of female fertility among the human and animal species symbolizes a paradox of evolution. Despite being born with a significant number of primordial cells which representing the ancestor cells of the germ-line, women experience a depletion of ovarian reserve and sub-fertility mid-way into their healthy lives, [14]. The varied rates at which body tissues aging increased, raise questions about the ongoing biological mechanisms which are involved in the regulation of aging in the germ and somatic cell lineages of the developing gonads. The ovaries are equally complex where regulation of follicles and oocytes become scarce during the course of the entire reproductive span that gives an intricate interplay between rates of induction, development, growth and finally follicular atresia [15].

Our study cohort comprises of infertile women (aged 25–39 years) who did not show any significant changes in the ovarian reserve estimated by AFC and AMH serum concentration compared with the controls of the same age groups. Moreover, the age-specific decline in the ratio of serum concentration of AMH to AFC was similar between the two cohorts, while the poor ovarian reserve, regardless of the cut-off level employed, was not over-represented in infertile women. Remarkably, our ovarian reserve data also revealed that the follicular dynamics were no longer significantly changed by improving sexual life when compared with the controls.

The distribution of follicle sizes in the infertile patients displayed that older women (36–39 years) had a high percentage of large follicles and lower levels of small follicles coupled with the slightly elevated serum concentration of FSH in comparison to the controls. In agreement with our results, a previous study revealed similar levels of FSH and AFC in the infertile and fertile women aged between 35 to 45 years [16]. However, another study demonstrated an abrupt age-specific decline in the serum concentration of AMH in the 197 infertile women (aged 19–47 years) compared to controls of a similar age [17]. Moreover, the infertile patients recruited in this previous study had a hormonal profile consistent with the menopausal transition of AMH concentration that decreased gradually, while FSH concentration steadily increased with increasing age and was attributed to poor ovarian reserve, therefore not consistent with the selection of the infertile cohort of our study [17].

Additionally, our results are in line with another study that found the similar mean ovarian volume, AFC, and FSH in both patient and control groups including 62 sterile women with unexplained infertility and 53 fertile women of age 35–45 years [18]. Our ovarian reserve data had shown that male infertility was associated with a high concentration of serum AMH and AFC in the infertile women below the age of thirty. The pattern of this association was identical after excluding the male-related genetic factors [19]. Subsequently, we did not find any existing link between poor ovarian reserve and unexplained infertility.

Together AMH and AFC are both quantitative markers of the ovarian stock in lieu of the available number of developing basal antral follicles. The AMH concentration in the blood indirectly measures the total mass of AMH-producing granulosa cells, while AFC comprises only antral follicles that are about > 2 mm in size. However, significant contribution to circulating AMH per follicle seems to be derived from follicles of size 5–8 mm [20]. Likewise, the relationship between AMH and AFC in terms of a pregnancy outcome remains unpredictable. Furthermore, natural and treatment-related therapeutic pregnancies do occur in women with poor ovarian reserve. Previously, research on women aged 35–45 years having AMH levels < 1.4 ng/ml demonstrated an age-specific decline in ovarian reserve, but there was no association found in young fertile women regarding decreased serum AMH levels and reduced fecundity [21, 22]. Accordingly, our results supported the fact that the available number of developing antral follicles and their ability to produce AMH during primary folliculogenesis was identical in both infertile women and controls. It is not possible to rule out that infertility might be instigated by a low oocyte quality regardless of follicular stock in certain patients. The tubal blockage is another primary cause of infertility, and infertile women are often screened for endometriosis associated with decreased follicular ovarian reserve markers [23]. However, it is not unexpected that the two cohorts were distinct in these aspects.

Even though the demand for infertility treatment has gradually increased in the last decade, but still the high cost may deter some couples from seeking care. The ratio of moderate to high-income patients finding the cure for infertility was high in all age groups as compared to low-income people. In particular, our findings also indicated that income has a most definite link with infertility treatments, as the probability of undergoing treatments were lowest among infertile patients with a monthly household income of about 30,000–50,000 rupees (RS). Thus, affordable IVF treatment would provide a framework from which to improve the ability of infertile patients to seek low-cost infertility assistance, choose better effective therapies, and accomplish their goal to have a family [24].

In the present study, the reported prevalence of heavy bleeding increased by 8% in women over 29 years is similar to the findings from other studies [25, 26]. The significant strength of our research findings is a comparatively good quality sample size and patients of considerable proportions who experienced a detailed examination and data collection. In some of the age-specific subclasses, the analysis revealed lower numbers of subjects which might be deficient in significant differences. Both control and patient groups were employed during the same period with a maximum of a one-year time interval. Moreover, similar laboratory equipment and ultrasound apparatus were used at the same center together with a similar algorithm.

The critical limitations of our study finding are I) the primary outcome that was conception not live birth, the similarly poor ovarian reserve may lead to high risk for miscarriage and abortion II) ovulation was not evaluated in the context of fecundability that is to say the ability to conceive and carry a fetus till birth. III) menstrual blood loss was assessed through face to face interviews and through objective measurement of menstrual blood loss but was not done through pictorial blood loss assessment chart.

## Conclusion

In conclusion, our results showed that antral follicle count and anti-Müllerian hormone showing similar trend irrespective of fertility status and menstrual characteristics in women under the age of 40 years. Hence, the possible common observation of low respondent in ART might not be a result of over-representation of patients with an early age-specific decline in the ovarian reserve, but rather a consequence of age-specific depletion in the stock of developing follicles at the time of recruitment and selection.

## Abbreviations

AFC: Antral follicle count
AMH: Anti-Müllerian hormone
ART: Assisted Reproductive Technology
PCOS: Polycystic Ovarian Syndrome

## References

[1] Boivin J, Bunting L, Collins JA, Nygren KG (2007). International estimates of infertility prevalence and treatment-seeking: potential need and demand for infertility medical care. Hum Reprod.

[2] Asemota OA, Klatsky P. Access to infertility care in the developing world: the family promotion gap. In: Seminars in reproductive medicine. New York: Thieme Medical Publishers; 2015. p. 017–22. 10.1055/s-0034-1395274 25565507

[3] Li HWR, Cheung TM, Yeung WSB, Ho PC, Ng EHY (2017). Relative importance of the different components of the Bologna criteria for predicting poor ovarian response in assisted reproduction. Maturitas.

[4] Shahine LK, Marshall L, Lamb JD, Hickok LR (2016). Higher rates of aneuploidy in blastocysts and higher risk of no embryo transfer in recurrent pregnancy loss patients with diminished ovarian reserve undergoing in vitro fertilization. Fertil Steril.

[5] Perry JR, McMahon G, Day FR, Ring SM, Nelson SM, Lawlor DA (2015). Genome-wide association study identifies common and low-frequency variants at the AMH gene locus that strongly predict serum AMH levels in males. Hum Mol Genet.

[6] Te Velde ER, Pearson PL (2002). The variability of female reproductive ageing. Hum Reprod Update.

[7] Hendriks DJ, Mol B-WJ, Bancsi LF, te Velde ER, Broekmans FJ (2005). Antral follicle count in the prediction of poor ovarian response and pregnancy after in vitro fertilization: a meta-analysis and comparison with basal follicle-stimulating hormone level. Fertil Steril.

[8] Depmann M, Broer SL, van der Schouw YT, Tehrani FR, Eijkemans MJ, Mol BW, Broekmans FJ (2016). Can we predict age at natural menopause using ovarian reserve tests or mother's age at menopause? A systematic literature review. Menopause.

[9] Medicine PCotASfR (2015). Testing and interpreting measures of ovarian reserve: a committee opinion. Fertil Steril.

[10] Gizzo S, Andrisani A, Noventa M, Quaranta M, Esposito F, Armanini D, Gangemi M, Nardelli GB, Litta P, D’Antona D (2015). Menstrual cycle length: a surrogate measure of reproductive health capable of improving the accuracy of biochemical/sonographical ovarian reserve test in estimating the reproductive chances of women referred to ART. Reprod Biol Endocrinol.

[11] Mutlu MF, Erdem M, Erdem A, Yildiz S, Mutlu I, Arisoy O, Oktem M (2013). Antral follicle count determines poor ovarian response better than anti-Müllerian hormone but age is the only predictor for live birth in in vitro fertilization cycles. J Assist Reprod Genet.

[12] Broer S, Eijkemans M, Scheffer G, Van Rooij I, De Vet A, Themmen A, Laven J, De Jong F, Te Velde E, Fauser B (2011). Anti-Müllerian hormone predicts menopause: a long-term follow-up study in normoovulatory women. J Clin Endocrinol Metab.

[13] Steiner AZ, Herring AH, Kesner JS, Meadows JW, Stanczyk FZ, Hoberman S, Baird DD. Antimüllerian hormone as a predictor of natural fecundability in women aged 30–42 years. Obstet Gynecol. 2011;117:132-45. 10.1097/AOG.0b013e3182116bc8 PMC3825553 21422850

[14] Hansen KR, Knowlton NS, Thyer AC, Charleston JS, Soules MR, Klein NA (2008). A new model of reproductive aging: the decline in ovarian non-growing follicle number from birth to menopause. Hum Reprod.

[15] Gougeon A (1996). Regulation of ovarian follicular development in primates: facts and hypotheses. Endocr Rev.

[16] Iliodromiti S, Anderson RA, Nelson SM (2014). Technical and performance characteristics of anti-Müllerian hormone and antral follicle count as biomarkers of ovarian response. Hum Reprod Update.

[17] Raeissi A, Torki A, Moradi A, Mousavipoor SM, Pirani MD (2015). Age-specific serum anti-mullerian hormone and follicle stimulating hormone concentrations in infertile Iranian women. Int J Fertil Steril.

[18] Erdem M, Erdem A, Biberoglu K, Arslan M (2003). Age-related changes in ovarian volume, antral follicle counts and basal follicle stimulating hormone levels: comparison between fertile and infertile women. Gynecol Endocrinol.

[19] Khan HL, Bhatti S, Abbas S, Khan YL, Aslamkhan M, Gonzalez RMM, Gonzalez GR, Aydin HH, Trinidad MS (2018). Trinucleotide consortium of androgen receptor is associated with low serum FSH and testosterone in asthenospermic men. Syst Biol Reprod Med.

[20] Jeppesen J, Anderson R, Kelsey T, Christiansen SL, Kristensen S, Jayaprakasan K, Raine-Fenning N, Campbell B, Yding Andersen C (2013). Which follicles make the most anti-Müllerian hormone in humans? Evidence for an abrupt decline in AMH production at the time of follicle selection. MHR: Basic Sci Reprod Med.

[21] Wilkosz P, Greggains GD, Tanbo TG, Fedorcsak P (2014). Female reproductive decline is determined by remaining ovarian reserve and age. PLoS One.

[22] Depmann M, Broer S, Eijkemans M, van Rooij I, Scheffer G, Heimensem J, Mol B, Broekmans F (2017). Anti-Müllerian hormone does not predict time to pregnancy: results of a prospective cohort study. Gynecol Endocrinol.

[23] Lemos NA, Arbo E, Scalco R, Weiler E, Rosa V, Cunha-Filho JS (2008). Decreased anti-Müllerian hormone and altered ovarian follicular cohort in infertile patients with mild/minimal endometriosis. Fertil Steril.

[24] Plante BJ, Cooper GS, Baird DD, Steiner AZ (2010). The impact of smoking on antimüllerian hormone levels in women aged 38 to 50 years. Menopause (New York, NY).

[25] Shapley M, Jordan K, Croft PR (2004). An epidemiological survey of symptoms of menstrual loss in the community. Br J Gen Pract.

[26] Janssen CA, Scholten PC, Heintz APM (1997). Menorrhagia—a search for epidemiological risk markers. Maturitas.

